# Spinal epidural involvement in adult Langerhans cell histiocytosis (LCH): A case report

**DOI:** 10.1097/MD.0000000000018794

**Published:** 2020-01-17

**Authors:** Cheong-Su Lim, Jae Hwan Cho

**Affiliations:** Department of Orthopedic Surgery, Asan Medical Center, University of Ulsan College of Medicine, Seoul, Korea.

**Keywords:** Langerhans cell histiocytosis, spinal cord compression, tuberculosis

## Abstract

**Rationale:**

Spinal involvement in adult Langerhans cell histiocytosis (LCH) is rare, and epidural involvement is unusual. LCH is mostly indistinguishable from other spinal lesions such as infection, lymphoma, and metastasis. So, it could be easily misdiagnosed without suspicion.

**Patient concerns:**

We report a case of a 33-year-old man who complained of gait disturbance with weakness in both legs and severe back pain.

**Diagnoses:**

A continuous enhancing epidural lesion with cord compression from the T7 to L1 level was detected in magnetic resonance imaging. Laboratory analysis indicated the possibility of spinal infectious disease. We assumed that the lesion could be tuberculous spondylitis.

**Interventions and outcomes:**

The patient underwent posterior laminectomy with marginal excision of the epidural mass to relieve cord compression. Pathological examination confirmed the diagnosis of LCH. The 12-month follow-up evaluation revealed that the patient was neurologically intact and had no gait disturbance.

**Lessons:**

This case report presents a patient with epidural LCH of the thoracic spinal cord, which can mimic spinal infections such as tuberculous spondylitis with abscess formation. Therefore, LCH could be considered as a possible diagnosis when a patient presents with features of infectious spondylitis with vertebral involvement.

## Abstract 2

Supplemental Digital Content is available in the text

## 1 Introduction

Langerhans cell histiocytosis (LCH) is associated with the clonal proliferation of Langerhans cells occurring as an isolated lesion or as part of a systemic (multifocal) proliferation.^[1]^ LCH is a rare disorder characterized by the excessive proliferation of pathologic Langerhans cells.^[1]^ The disease varies widely in clinical presentation from the localized involvement of a single bone to a widely disseminated life-threatening disease.^[2]^ Spinal involvement in adult LCH is rare, and epidural involvement is unusual.^[3–5]^ LCH is mostly indistinguishable from other spinal lesions such as infection, lymphoma, and metastasis. Here, we report a case of a 33-year-old man diagnosed with epidural LCH of the thoracic spinal cord, which was confusing as clinical, laboratory, imaging findings indicated a spinal inflammatory disease.

## 2 Case report

Informed written consent was obtained from the patient for publication of this case report and accompanying images

A 33-year-old man was presented with 1 year of lower thoracic back pain, 7 days of gait disturbance, and weakness in both lower extremities. He also complained that he had difficulty maintaining his balance. There was no history of systemic illness and family history. On examination, the deep tendon reflex of both knees was exaggerated, and the motor grade of both lower extremities (hip flexion only, grade 4) was decreased. Blood test variables including white blood cells (WBC), erythrocyte sedimentation rate (ESR), and C-reactive protein (CRP) were increased (WBC, 10.6 K/μl [4 K/μl–10 K/μl]; ESR, 48 mm/hour [0–28 mm/hour]; CRP, 5.10 mg/dl [0–0.3 mg/dl]). His body temperature was normal, and there was no inflammatory focus in other systems on clinical examination.

**Figure 1:**
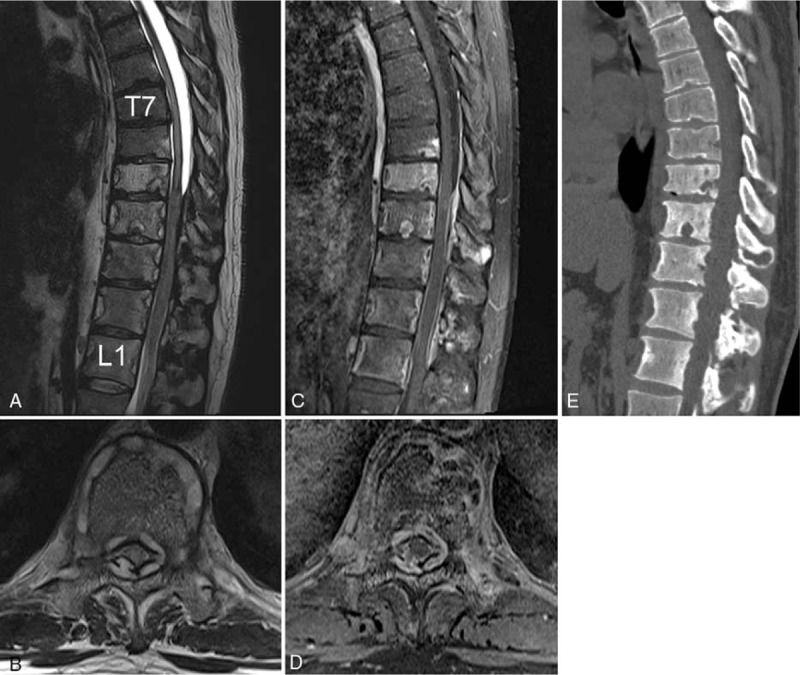
Preoperative T2-weighted (A and B), T1 gadolinium-enhanced (C and D) sagittal MR images, and axial images at the level of T10, (E) a sagittal CT reconstructive image showing a homogeneous continuous enhancing epidural lesion and osteolytic bone lesion at the T7–L1 level.

**Figure 2:**
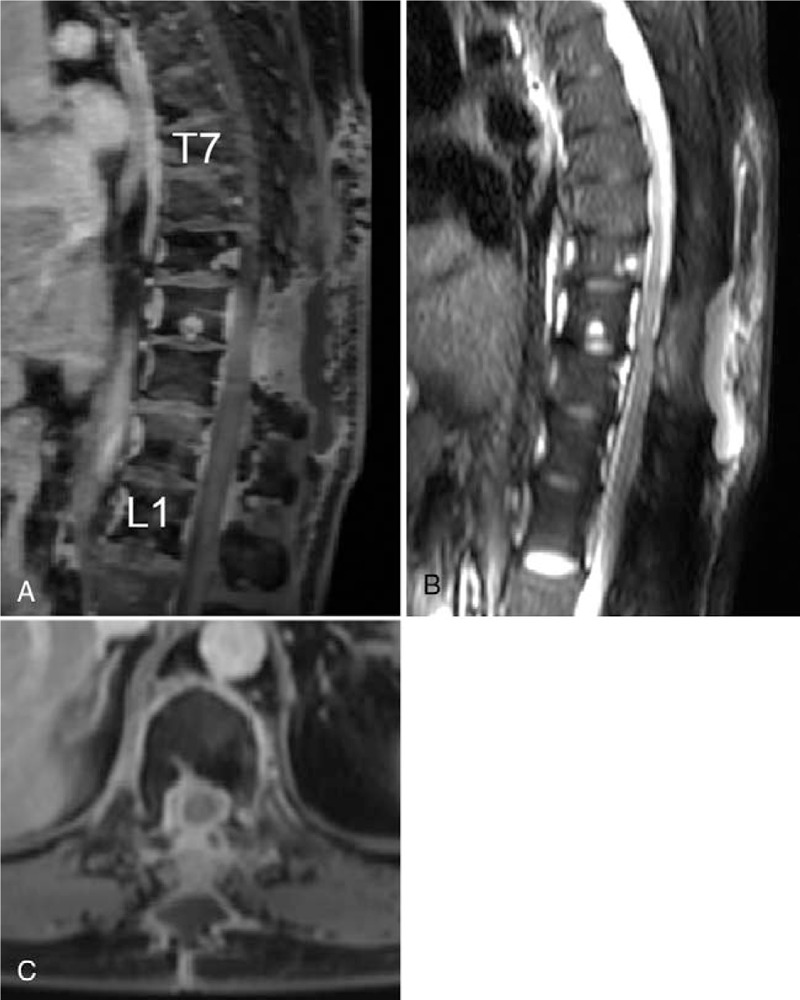
Preoperative T1 gadolinium-enhanced (A and B), T2-weighted (C) sagittal MR images, and axial images at the level of T10 showing a decompression of the cord and remaining mass lesion.

Magnetic resonance imaging (MRI) showed a continuous enhancing epidural lesion, and computed tomography (CT) showed multiple osteolytic bone lesions at the T7–L1 level with spinal cord compression at the T9–T12 level (Fig. 1). Therefore, we assumed that the lesion was infectious spondylitis with epidural phlegmon and abscess, such as tuberculous infection. As there were gait disturbance, motor weakness, and cord compression, the patient underwent posterior laminectomy (T9–10 total laminectomy—most severe compressed level) with marginal excision of the epidural mass (Fig. 2). The epidural mass could be removed completely because it was sticky and adhered to the dura mater (Supplementary 1).

**Figure 3:**
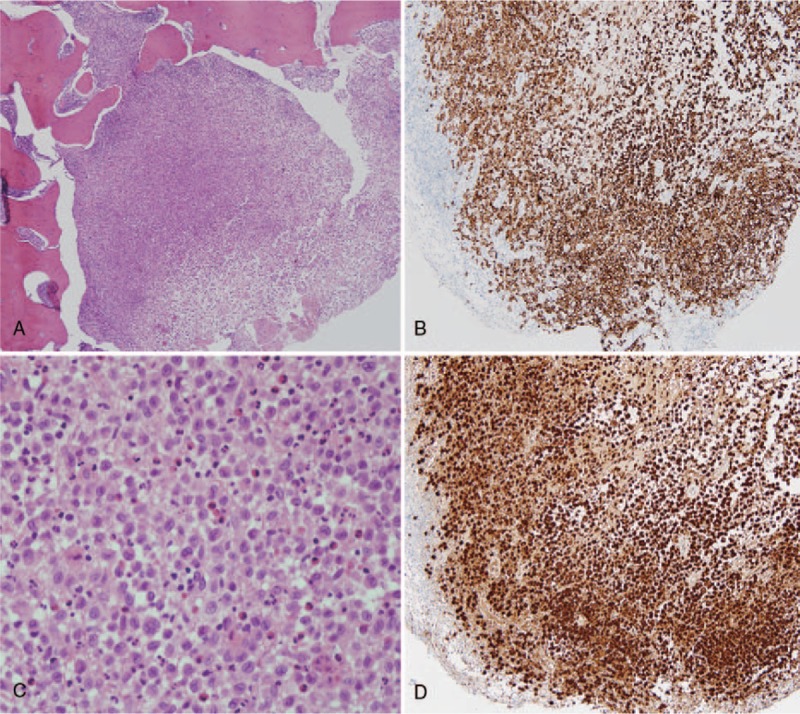
Photomicrographs of the epidural lesion. The tumor is composed of a large number of B cells and plasma cells. (A) H&E staining, X40, (B) H&E staining, X400, (C) CD1a staining, and (D) S-100 immunohistochemical staining.

**Figure 4:**
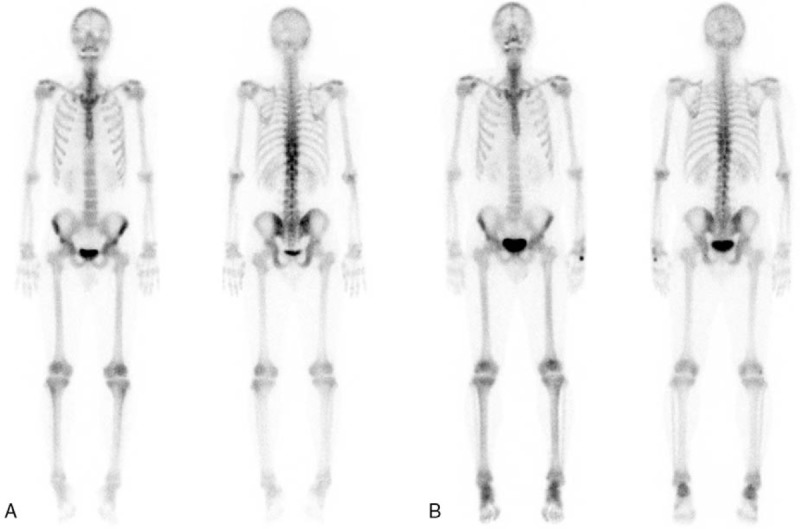
A comparison of bone scan images between the (A) preoperative and (B) postoperative 1-year period.

Pathological examination confirmed the diagnosis of LCH (Fig. 3). There was a positive immunohistochemical reaction with the CD1a and S-100 protein. After surgery, the patient showed a progressive improvement in gait disturbance. Subsequently, he underwent chemotherapy with prednisolone and vinblastine for 10 months. The 12-month follow-up evaluation revealed that the patient was neurologically intact and had no gait disturbance. The follow-up bone scan obtained at the end of chemotherapy showed decreased LCH (Fig. 4).

## 3 Discussion

LCH is a rare disease with a rather unpredictable course as it can spontaneously resolve or progress to a disseminated form, compromising vital functions with occasionally fatal consequences. LCH is diagnosed mostly in children and adolescents and rarely in adults. The peak incidence is between the ages of 5 and 10 years in about 80% of cases.^[6,7]^ The incidence of LCH in adults may be as high as 1 to 2 cases per million.^[8]^ Among adult patients older than 18 years old, multiple system involvement (69%) is more common than single system involvement (31%) at the time of diagnosis. In single system involvement, LCH mainly involves the lungs (51.1%), bone (38.3%), and skin (7.01%).^[9]^ In cases involving the bone, the location of lesions includes the skull (27%), femur (15%), and spine (6.5%).^[10]^ In our case, the patient was diagnosed with LCH as an adult, and the lesions involved the spine but not the lungs, skull, and appendicular skeleton.

Vertebral lesions occur most commonly in the thoracic spine and less often in the lumbar and cervical spine.^[11]^ The lesions are usually solitary, and the vertebral bodies are the main structures affected.^[3,4,6,12]^ The most common radiographic features in spinal LCH are lytic lesions of the vertebra, which can usually lead to vertebral collapse.^[5]^ In our case, there were altered signal intensities with enhancement in the vertebral bodies; however, the vertebral height was relatively intact. MRI revealed a continuous enhancing epidural lesion with spinal cord compression. Huang et al have reported that the typical signs of LCH are seldom observed in adults (11%), and epidural cord compression with neurologic symptoms is more common in adults than in children.^[4]^ The relatively preserved disc space as well as the multiplicity of vertebral body involvement and paraspinal and epidural enhanced lesions may suggest the possibility of tuberculous spondylitis.^[13–16]^

In our case, WBC, ESR, and CRP were sufficiently elevated to suggest the possibility of spinal infectious disease. Hence, we wrongly assumed that the lesion could be tuberculous spondylitis based on MRI findings and laboratory results. It is well known that LCH involvement can lead to the elevation of inflammatory markers such as WBC, ESR, and CRP. However, in previous studies related to adult LCH, results on inflammatory markers were absent or reported as normal.^[5,6,12,17]^

The patient underwent posterior laminectomy with marginal excision of the epidural mass to relieve cord compression. The preoperative impression was tuberculous spondylitis with abscess formation; however, intraoperative findings did not correspond to an infection. The epidural mass had a whitish color and hard rubber-like consistency. Pathological examination confirmed the diagnosis of LCH. Similar to our case, the misdiagnosis between tuberculosis and LCH has been reported in some cases involving children.^[18,19]^ To the best of our knowledge, there are no such reports for adults.

In MRI, epidural LCH is usually indistinguishable from other spinal lesions such as infection, lymphoma, and metastasis. Laboratory and clinical findings often lead to an inaccurate differential diagnosis. Ultimately, a definite diagnosis can be established by pathological confirmation. Therefore, diagnosis and treatment should not be concluded before evaluating biopsy results. Given the different possible causes, a diagnostic modality for tissue confirmation such as CT-guided biopsy should be performed.

The optimal treatment strategy for spinal LCH remains controversial, and several treatment approaches including bed rest, immobilization with cast and brace, chemotherapy, radiation therapy, and surgery have been proposed. Nevertheless, the appropriate treatment options for adults have not been clarified by clinical trials and the literature. Some recommendations have been proposed for the evaluation and treatment of adult patients with LCH.^[20]^ Chemotherapy may be used as a first-line therapy for patients without neurological deficits or as an adjuvant therapy after surgery, especially in cases with multiple bone lesions such as in the present case. Patients with evident signs of spinal cord compression, such as in our case, should promptly undergo surgical decompression to improve the chances of neurological recovery and to obtain tissues for histopathological examination. Regarding an optimal first-line chemotherapy agent, some experienced clinicians prefer to start with vinblastine and prednisone. However, vinblastine and prednisone have not been proven effective for adults in a prospective study. Due to a lower risk of neurotoxicity and unacceptable steroid-induced side effects, some experts prefer monotherapy with cladribine, cytarabine, or etoposide.^[20]^ In our case, the patient underwent surgical decompression and postoperative chemotherapy with vinblastine and prednisone.

In summary, we report a case of adult epidural LCH with spinal cord compression mimicking spinal abscess due to Mycobacterium tuberculosis. Although extremely rare, LCH should be considered when there are epidural lesions with spinal cord compression in adults.

## Author contributions

**Conceptualization:** Jae Hwan Cho.

**Data curation:** Cheong-Su Lim.

**Formal analysis:** Cheong-Su Lim.

**Investigation:** Cheong-Su Lim.

**Methodology:** Jae Hwan Cho.

**Supervision:** Jae Hwan Cho.

**Validation:** Jae Hwan Cho.

**Writing – original draft:** Cheong-Su Lim.

**Writing – review & editing:** Jae Hwan Cho.

## Supplementary Material

Supplemental Digital Content
